# Supplementary material for: Early succession on slag compared to urban soil: A slower recovery
Source: PLoS One. 2019 Dec 19;14(12):e0224214. doi: 10.1371/journal.pone.0224214 (PMC6922358; doi:10.1371/journal.pone.0224214)
Supplement: S1 File — Detailed history of management for both Reference sites and methods for data analysis, including the list of R packages used. All supplemental results (Table A-J, Fig A-E and all associated captions) are also included. A glossary for site, plot and treatment numbering is included. (DOCX) [file pone.0224214.s001.docx]

**Supporting Information**

For

**Early Succession on Slag Compared to Urban Soil: A Slower Recovery**

Heng-Xing Zou, Alison E. Anastasio, Catherine A. Pfister

**Supplementary Methods**

All data analyses and plotting were done in R (R Core Team 2013) using packages *ade4* (Dray and Dufour 2007), *cluster* (Maechler et al. 2018), *dendextend* (Galili 2015), *factoextra* (Kassambara and Mundt 2017), *FD* (Laliberté and Legendre 2010), *ggvegan* (Simpson 2017), *lindia* (Lee and Ventura 2017), *nlme* (Pinheiro et al. 2018), *PCAmixdata* (Chavent et al. 2017), *tidyverse* (Wickham 2017) and *vegan* (Oksanen et al. 2018).

*Past Management and Plant Survey on Reference Sites*

The two Reference sites, BM-R and VV-R, consist of urban soil on construction debris of complex origins (Lauren Umek, Chicago Park District, *personal communication*). Active management has taken place at both sites. BM-R was managed for invasive species (aerial herbicide and manual removal) in 2015 and 2016, and subsequently seeded by Chicago Park District in 2017 (see **S10 File** for complete list of species). VV-R received relatively fewer managements; instead of a full inventory, a rapid plant survey (methods unknown) was conducted, with resulting species list in **S10 File**. Both sites were mowed during our experimental period.

*Soil Test*

Two methods of transformation were performed on the original data set. Because sand, silt, and clay content were compositional measurements (that is, percentages added up to 1), one measurement was sequentially excluded from the analysis while the remaining two were log-transformed. In the second transformation, all measurements were log-transformed. Both transformations resulted in the same clustering pattern and same set of indicative variables; the PCA result from second transformation was shown to display all soil variables.

*Functional Trait Analysis*

To calculate a species distance matrix based on functional traits, which consisted of both quantitative and nominal data, two methods were used. First, a Gower’s distance (Gower 1971) was calculated using the function *gowdis* from R package *FD* (Laliberté and Legendre 2010). Second, a distance matrix was computed by the function *dist.ktab* from R package *ade4* (Dray and Dufour 2007) following Pavoine et al. (2009); the method takes two separate matrices of quantitative and nominal data, computes Gower’s distance matrices separately, then combines the two results. Species were clustered based on the distance matrix calculated by the latter method, using hierarchical clustering functions by the R package *factoextra* (Kassambara and Mundt 2017).

*Fourth Corner Analysis*

**R**, **L** and **Q** stand for matrices with environmental variables, species distribution (abundance or presence-absence) and functional trait information, respectively; the fourth-corner method was used to analytically detect correlations between those matrices (Dray and Legendre 2008). Specifically, the fourth-corner method permutes variable elements of community matrices with a Monte-Carlo test and compares the statistics of random versus observed matrices, detecting potential effects of environmental variables (Dray and Legendre 2008). For the purpose of this study, we permuted both site and species vectors to generate 1000 randomized presence-absence matrices. First, the significance of associations between each environmental variable and functional trait was evaluated using the function *fourthcorner*; then, the *S***_RLQ_** statistic (a multivariate statistic similar to the correlation coefficient; see Dray and Legendre 2008) was applied to the model using the function *fourthcorner2* to determine the overall association between environmental variables and functional traits.

*Topsoil Germination Assay*

We observed an overrepresentation of nonnative species in topsoil plots compared to removal plots suggesting potential contamination of topsoil. In fact, 11 nonnative species, including purslane (*Portulaca oleracea*), smooth crabgrass (*Digitaria ischaemum*) and redshank (*Persicaria maculosa*), were present in all topsoil plots but no removal plots. In order to identify whether seeds were latent in topsoil, we performed germination assays (in the greenhouse to avoid any possible propagules from outside) with topsoil only. Six species germinated from topsoil trays, indicating that our topsoil was contaminated by predisposed seeds. Since the germination assay including only a small subsample of the total amount of topsoil used, though only six species germinated in the greenhouse, this result suggests that the remaining five species found only in topsoil experimental plots may have also come from the topsoil. However, it does not exclude the possibility that there was differential germination of nonnative species from the local environment.

See **S3 File** for all species presence-absence data, including lists of species in the greenhouse germination assay and only present in topsoil plots. See also **S4** **File** for native status of species colonizing germination plots, determined by Hilty (2017), excluding species from the germination assay or only present in topsoil plots.

**Glossary**

Locale: Big Marsh (BM) or Van Vlissingen (VV)

Site: Slag (S) or Reference (R)

Block: Consist of either 3 (for 2 blocks at both Reference sites) or 5 (all else) plots of different purposes. Considered as replicates; numbered A-E.

Treatments: Removal (1) or topsoil (0).

Focal Species: Common milkweed (*Asclepias Syriaca*, AS), sideoats grama (*Bouteloua curtipendula*, BC) and showy goldenrod (*Solidago speciosa*, SS).

Full notation of a plot:

Locale-Site-Block-Treatment

e.g. BM-S-A-1 means removal plot of block A on Big Marsh Slag.

**Supplemental Results**

**Table A.** **Soil test results of samples from sites and commercial topsoil**. Two samples were obtained from VV-S, denoted as VV-S-1 and VV-S-2. All unit for elemental concentration is parts per million (ppm).

| Measurement | BM-R | VV-R | BM-S | VV-S-1 | VV-S-2 | Topsoil |
| --- | --- | --- | --- | --- | --- | --- |
| Ca | 2150 | 3850 | 10700 | 10150 | 9350 | 4800 |
| Mn | 36 | 59 | 225 | 109 | 143 | 16 |
| K | 106 | 175 | 108 | 258 | 281 | 75 |
| Zn | 18.9 | 41.4 | 70.6 | 10.5 | 26.5 | 2.9 |
| Cr | 20.5 | 35.3 | 227 | 15.6 | 19 | 6.10 |
| Sand (%) | 50 | 50 | 80 | 72 | 78 | 78 |
| CEC (meq/100 g) | 15.2 | 23.3 | 57.4 | 52.7 | 48.8 | 26.6 |
| pH | 7.9 | 7.2 | 9.2 | 8.2 | 8 | 7.4 |
| Cu | 6.3 | 9.9 | 3.6 | 1.9 | 2.7 | 1.1 |
| Pb | 79.4 | 353 | 147 | 38 | 181 | 8.36 |
| Mg | 505 | 430 | 435 | 160 | 165 | 285 |
| Clay (%) | 24 | 22 | 6 | 8 | 6 | 8 |
| Silt (%) | 26 | 28 | 14 | 20 | 16 | 14 |
| Organic Matter (%) | 6.1 | 6.6 | 5.8 | 2.3 | 3.4 | 18.0 |
| P (Bray, Total) | 18 | 20 | 22 | 3 | 3 | 32 |
| N (Total) | 8 | 21 | 33 | 5 | 8 | 72 |
| As | 9.8 | 7.99 | 6.11 | 3 | 3.63 | 15.7 |
| Fe | 68 | 42 | 51 | 13 | 53 | 89 |

**Table B.** **Plant functional traits and abbreviations used in S4 File File.**

| Trait Category | Trait Name | Abbreviations/Units |
| --- | --- | --- |
| Lifestyle | Species type | herb, grass, vine, shrub, tree (no abbreviation) |
|  | Life history | A: annual; B: biennial; P: perennial |
|  | Growth form | G: grass; TF: tall forb; SF: short forb; S: shrub; T: tree |
|  | Functional group | G: graminoid; F: forb; L: legume; W: woody |
| Physiology | Growth habit | E: erect; D: decumbent; P: procumbent; S: sprawling; V: vine |
|  | Shoot structure | L: leafy; S: semirosette; R: rosette |
|  | Canopy length | Quantitative, in cm |
| Regeneration | Regenerative strategy | W: widespread seed; V: vegetative spread; S: seasonal by seed |
|  | Seed number | Quantitative |
|  | Seed dry mass | Quantitative, in mg |
|  | Seedbank longevity | S: short (under 1 yr); M: medium (1 to 5 yrs); L: long (> 5 yrs) |
|  | Lateral spread | <0.01 m; 0.01-0.25 m; >0.25 m |
|  | Phenology | E: early (before June); S: summer (June to July); L: late summer (after July) |
| Primary Production | Leaf dry matter content (LDMC) | Quantitative, in mg/mg |
|  | Specific leaf area (SLA) | Quantitative, in mm^2^/mg |
| Native status | Native? | Y: native; N: nonnative |

|  | 7 | 14 | 21 | 28 | 35 | 42 | 49 | 56 | 63 | 70 | 77 | 84 | 91 | 98 | 105 | 112 |
| --- | --- | --- | --- | --- | --- | --- | --- | --- | --- | --- | --- | --- | --- | --- | --- | --- |
| BM Removal | **.007** | **.004** | **.036** | .090 | **.045** | .216 | .228 | .309 | **.018** | **.013** | .108 | .099 | .073 | .182 | .085 | **.013** |
| BM Topsoil | .354 | .186 | .201 | .596 | .667 | .187 | .0915 | .102 | **.012** | **<0.001** | **.005** | **<0.001** | **.020** | **.001** | **<0.001** | **<0.001** |
| VV Removal | .626 | .138 | .112 | .252 | .932 | .946 | **.005** | **.007** | **.001** | **<0.001** | **<0.001** | **<0.001** | **<0.001** | **<0.001** | **<0.001** | **<0.001** |
| VV Topsoil | .347 | .686 | .158 | .211 | .0638 | .169 | **.006** | **<0.001** | **<0.001** | **<0.001** | **<0.001** | **<0.001** | **<0.001** | **<0.001** | **<0.001** | **<0.001** |

**Table C.** ***p* value table of ANOVA on cover between Slag and Reference at each time point**. Rows: type of plots; columns: days since start. *p* values < 0.05 are considered significant, marked by bold.

**Table D.** ***p* value table of ANOVA on species number between Slag and Reference at each time point**. Rows: type of plots; columns: days since start. *p* values < 0.05 are considered significant, marked by bold.

|  | 7 | 14 | 21 | 28 | 35 | 42 | 49 | 56 | 63 | 70 | 77 | 84 | 91 | 98 | 105 | 112 |
| --- | --- | --- | --- | --- | --- | --- | --- | --- | --- | --- | --- | --- | --- | --- | --- | --- |
| BM Removal | **.007** | **.004** | **.036** | .090 | **.045** | .216 | .228 | .309 | **.018** | **.013** | .108 | .099 | .073 | .182 | .085 | **.013** |
| BM Topsoil | .354 | .186 | .201 | .596 | .667 | .187 | .0915 | .102 | **.012** | **<0.001** | **.005** | **<0.001** | **.020** | **.001** | **<0.001** | **<0.001** |
| VV Removal | .626 | .138 | .112 | .252 | .932 | .946 | **.005** | **.007** | **.001** | **<0.001** | **<0.001** | **<0.001** | **<0.001** | **<0.001** | **<0.001** | **<0.001** |
| VV Topsoil | .347 | .686 | .158 | .211 | .0638 | .169 | **.006** | **<0.001** | **<0.001** | **<0.001** | **<0.001** | **<0.001** | **<0.001** | **<0.001** | **<0.001** | **<0.001** |

**Table E.** **Linear regression results of species number on cover from removal plots**. *p* < 0.05 is considered significant, marked by bold.

|  | Intercept | Slope | *R*^2^ | *p* (slope) |
| --- | --- | --- | --- | --- |
| BM Slag | 7.065 | 0.609 | 0.054 | .385 |
| BM Reference | -18.386 | 4.399 | 0.909 | **<0.001** |
| VV Slag | -4.233 | 1.662 | .580 | **<0.001** |
| VV Reference | -20.161 | 5.104 | .570 | **<0.001** |

| Locale | Biomass, removal | Biomass, topsoil | Biomass, AS | | Biomass, BC | | Biomass, SS | |
| --- | --- | --- | --- | --- | --- | --- | --- | --- |
|  |  |  | Initial | Final | Initial | Final | Initial | Final |
| BM | **.009** | **.045** | .309 | .090 | .997 | **<0.001** | .478 | **<0.001** |
| VV | **.032** | **<0.001** | .899 | **.026** | .388 | **<0.001** | .855 | **<0.001** |

**Table F.** **Welch’s t-test results between Slag and Reference biomass measurements**. All biomass data were log-transformed; biomass of dead individuals was marked as 0. All tests on final harvest biomass (focal species and germination) were conducted with alternative hypothesis as “Reference biomass is larger than Slag biomass”; tests on initial harvest biomass were two-sided. *p* < 0.05 is considered significant, marked by bold.

**Table G.** **Two-way ANOVA on site and treatment effects of germination plot biomass**. An interaction term (Site × Treatment) was not included. Table shows *F* value with significance level notations: *p* > 0.05 (ns), *p* < 0.05 (*), 0.01 < *p* < 0.05 (**), *p* < 0.01 (***). Degree of freedom is 1 for both site and treatment.

| Locale | Site (Slag or Reference) | Treatment (Removal or Topsoil) |
| --- | --- | --- |
| BM | 21.335*** | 0.044 |
| VV | 33.53*** | 12.85** |

**Table H.** **Permutational Multivariate ANOVA (PERMANOVA) results for species presence-absence in removal and topsoil plots on different sites**. Site and block effects are individually tested. The test was conducted by function *adonis* in the R package *vegan* (Oksanen et al. 2018) on a Bray-Curtis distance matrix based on species presence-absence data in **S3 File**.

|  | Removal | | | Topsoil | | |
| --- | --- | --- | --- | --- | --- | --- |
|  | *F* | *R*^2^ | *p* value | *F* | *R*^2^ | *p* value |
| Site | *F*_3_ = 3.1158 | 0.726 | **0.001** | *F*_3_ = 2.903 | 0.463 | **0.001** |
| Block | *F*_4_ = 0.3003 | 0.070 | 0.834 | *F*_4_ = 0.524 | 0.111 | 0.983 |
| Residuals | (Deg. of Freedom = 8) | 0.204 | NA | (Deg. of Freedom = 8) | 0.425 | NA |

| Measurement | | Slope | | | | |
| --- | --- | --- | --- | --- | --- | --- |
|  |  | Cover | Biomass, germination | Biomass, AS | Biomass, BC | Biomass, SS |
| Slag | Ca | -.003 | -28.030 | -0.030 | -3.068 | -2.253 |
|  | pH | -12.370 | -191.120 | -0.596 | -41.260 | -14.55 |
|  | Sand | -0.374 | -23.070 | -0.179 | -14.575 | -7.240 |
|  | CEC | -0.609 | -34.770 | -0.041 | -3.949 | -2.755 |
|  | Mn | -0.099 | -13.970 | -0.035 | -2.908 | -2.313 |
|  | Cr | -0.062 | -7.969 | -0.013 | -0.505 | -1.021 |
|  | K | -0.076 | -23.270 | 0.057 | 1.335 | -0.438 |
|  | Zn | -0.129 | -7.014 | 0.015 | 1.832 | -0.771 |
| Reference | Silt | 0.529 | -1.044 | 0.153 | 11.126 | 5.483 |
|  | Clay | 0.837 | 15.862 | 0.058 | 4.823 | 2.543 |
|  | Mg | 0.027 | 15.51 | 0.022 | 3.675 | 1.254 |
|  | Cu | 1.256 | -3.078 | 0.069 | 5.923 | 1.686 |
|  | Pb | 0.023 | -4.567 | 0.055 | 4.398 | 0.253 |
| Topsoil | P (Bray, total) | 0.650 | 15.764 | 0.016 | 2.274 | 0.385 |
|  | N (Total) | 0.177 | 11.061 | 0.019 | 2.029 | -0.788 |
|  | Organic Matter | 0.914 | 18.425 | 0.005 | 5.425 | 1.269 |
|  | Fe | 0.207 | 19.165 | 0.002 | 1.750 | 0.901 |
|  | As | 1.367 | 24.640 | 0.038 | 4.782 | 2.168 |

**Table J.** **Linear regression results of soil and plant growth**. Negative slopes are marked with blue, positive with orange. Note that most measurements characterizing slag are negatively correlated with growth measurements. *p* < 0.003125 is considered significant, marked by bold.

**Table J (Continued)**

| Measurement | | *R^2^* | | | | | *p* value | | | | |
| --- | --- | --- | --- | --- | --- | --- | --- | --- | --- | --- | --- |
|  |  | Cover | Biomass, germination | Biomass, AS | Biomass, BC | Biomass, SS | Cover | Biomass, germination | Biomass, AS | Biomass, BC | Biomass, SS |
| Slag | Ca | 0.241 | 0.126 | 0.018 | 0.152 | 0.456 | 0.004 | 0.046 | 0.233 | **<0.001** | **<0.001** |
|  | pH | 0.222 | 0.169 | 0.118 | 0.464 | 0.321 | 0.006 | 0.020 | **0.001** | **<0.001** | **<0.001** |
|  | Sand | 0.052 | 0.011 | 0.062 | 0.337 | 0.463 | 0.208 | 0.568 | 0.025 | **<0.001** | **<0.001** |
|  | CEC | 0.239 | 0.143 | 0.022 | 0.171 | 0.464 | 0.004 | 0.033 | 0.185 | **<0.001** | **<0.001** |
|  | Mn | 0.173 | 0.143 | 0.152 | 0.140 | 0.495 | 0.018 | 0.033 | 0.164 | **<0.001** | **<0.001** |
|  | Cr | 0.074 | 0.072 | 0.008 | 0.010 | 0.225 | 0.133 | 0.138 | 0.442 | 0.098 | **<0.001** |
|  | K | 0.082 | 0.078 | 0.019 | 0.009 | 0.005 | 0.112 | 0.122 | 0.223 | 0.122 | 0.007 |
|  | Zn | 0.030 | 0.054 | 0.005 | 0.063 | 0.062 | 0.341 | 0.201 | 0.525 | **<0.001** | **<0.001** |
| Reference | Silt | 0.023 | <0.001 | 0.075 | 0.322 | 0.436 | 0.412 | 0.969 | 0.014 | **<0.001** | **<0.001** |
|  | Clay | 0.081 | 0.035 | 0.054 | 0.304 | 0.471 | **0.003** | 0.307 | 0.037 | **<0.001** | **<0.001** |
|  | Mg | 0.026 | 0.020 | 0.004 | 0.100 | 0.065 | 0.381 | 0.436 | 0.567 | **<0.001** | **<0.001** |
|  | Cu | 0.037 | 0.004 | 0.078 | 0.473 | 0.214 | 0.293 | 0.073 | 0.012 | **<0.001** | **<0.001** |
|  | Pb | 0.017 | 0.027 | 0.086 | 0.457 | 0.008 | 0.471 | 0.367 | 0.008 | **<0.001** | 0.127 |
| Topsoil | P (Bray, total) | 0.139 | 0.116 | 0.008 | 0.124 | 0.020 | 0.035 | 0.057 | 0.433 | **<0.001** | 0.019 |
|  | N (Total) | 0.079 | 0.090 | 0.009 | 0.082 | 0.069 | 0.119 | 0.095 | 0.400 | **<0.001** | **<0.001** |
|  | Organic Matter | 0.114 | 0.138 | 0.017 | 0.191 | 0.058 | 0.060 | 0.037 | 0.243 | **<0.001** | **<0.001** |
|  | Fe | 0.095 | 0.116 | <0.001 | 0.043 | 0.064 | 0.086 | 0.057 | 0.945 | **<0.001** | **<0.001** |
|  | As | 0.148 | 0.155 | 0.012 | 0.162 | 0.19 | 0.030 | 0.026 | 0.324 | **<0.001** | **<0.001** |


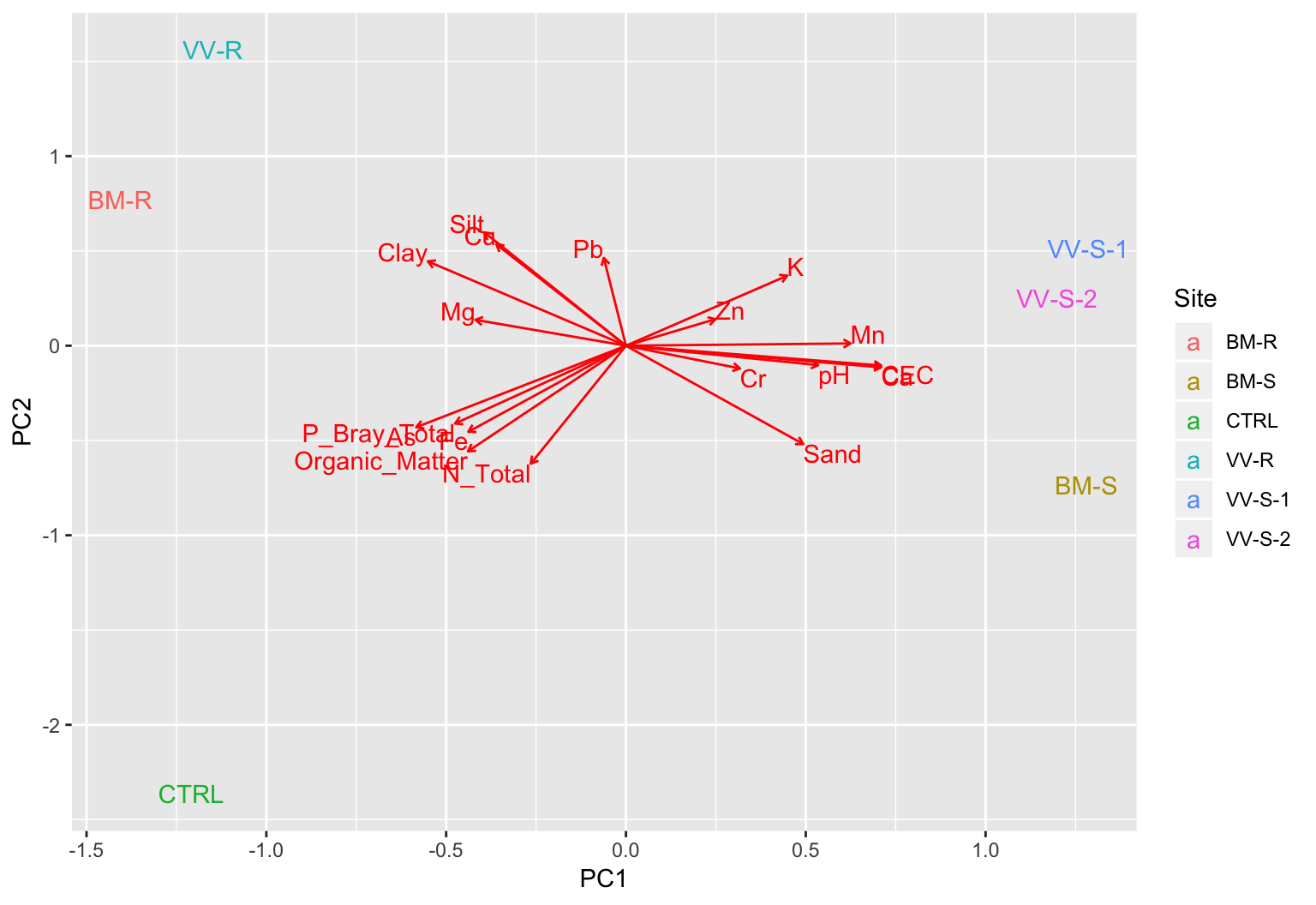


**Fig A.** **PCA analysis on soil data.**

Sites are color coded and red arrows showing vectors of soil measurements. See **Table A** for complete list of soil measurements.


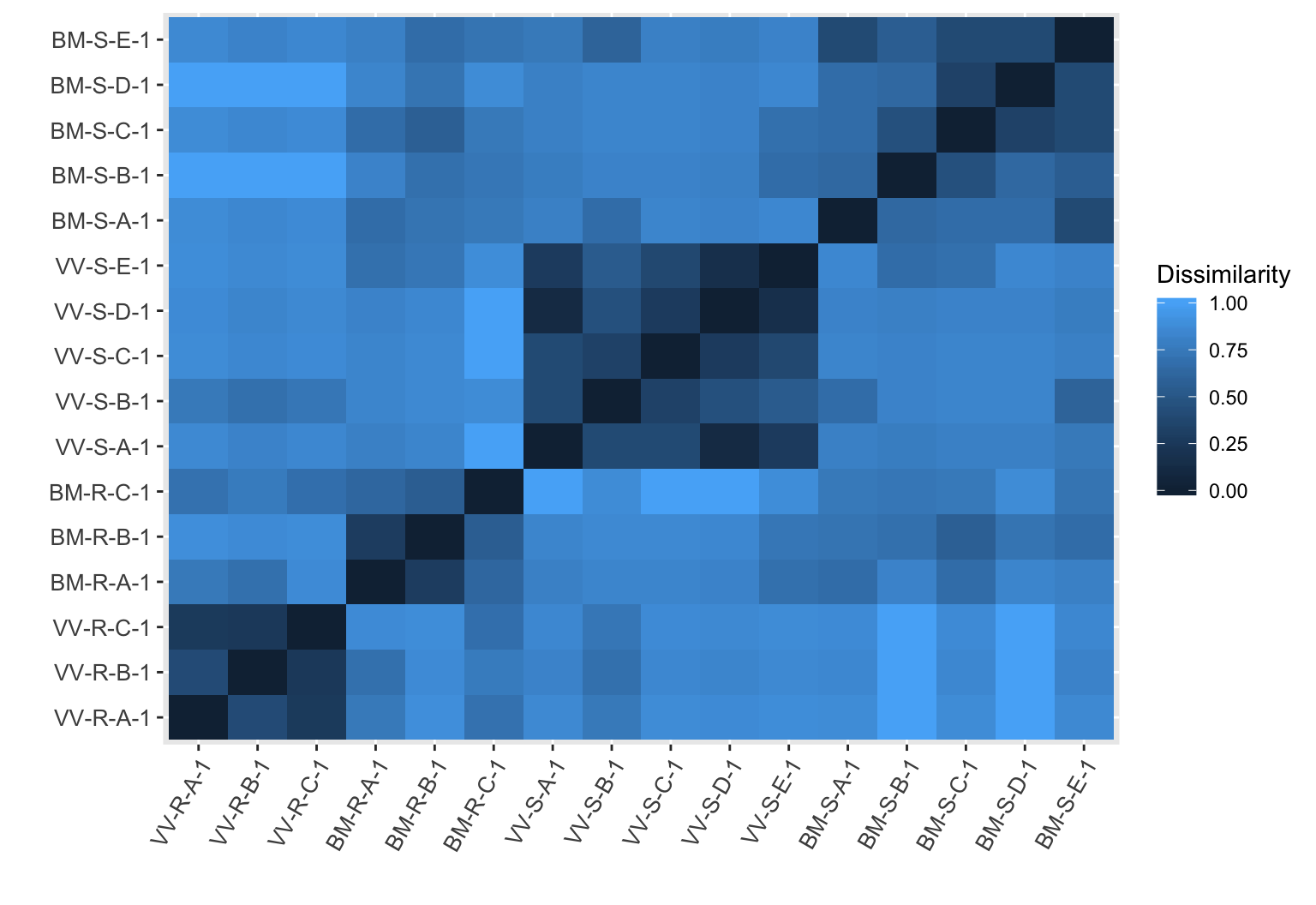


**I**


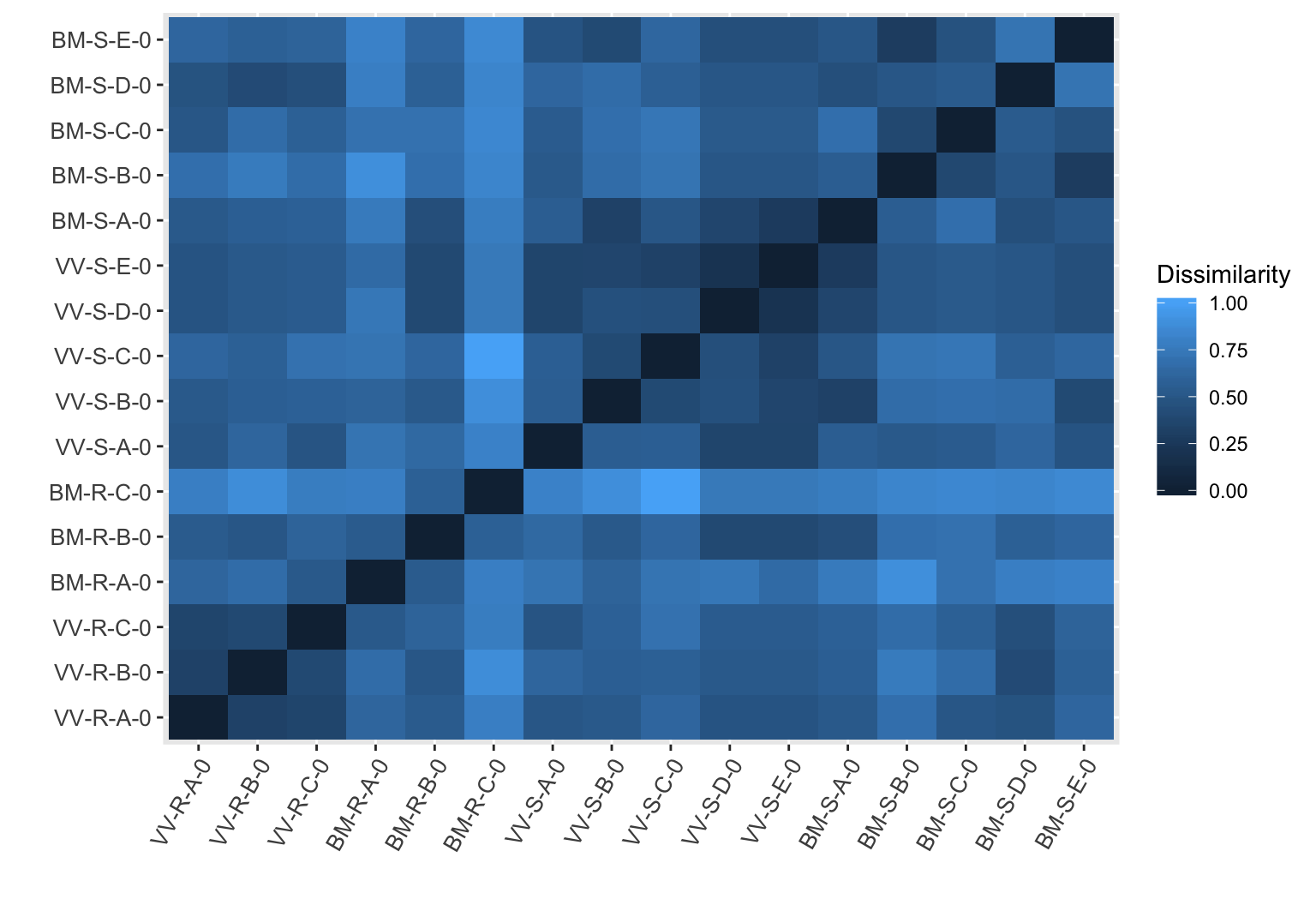


**II**

**Fig B. Heatmap of germination plots dissimilarity.**

(I) Removal plots. (II) Topsoil plots. Dissimilarity indices were calculated as Whittaker’s *β* (Koleff et al. 2003).

**III**


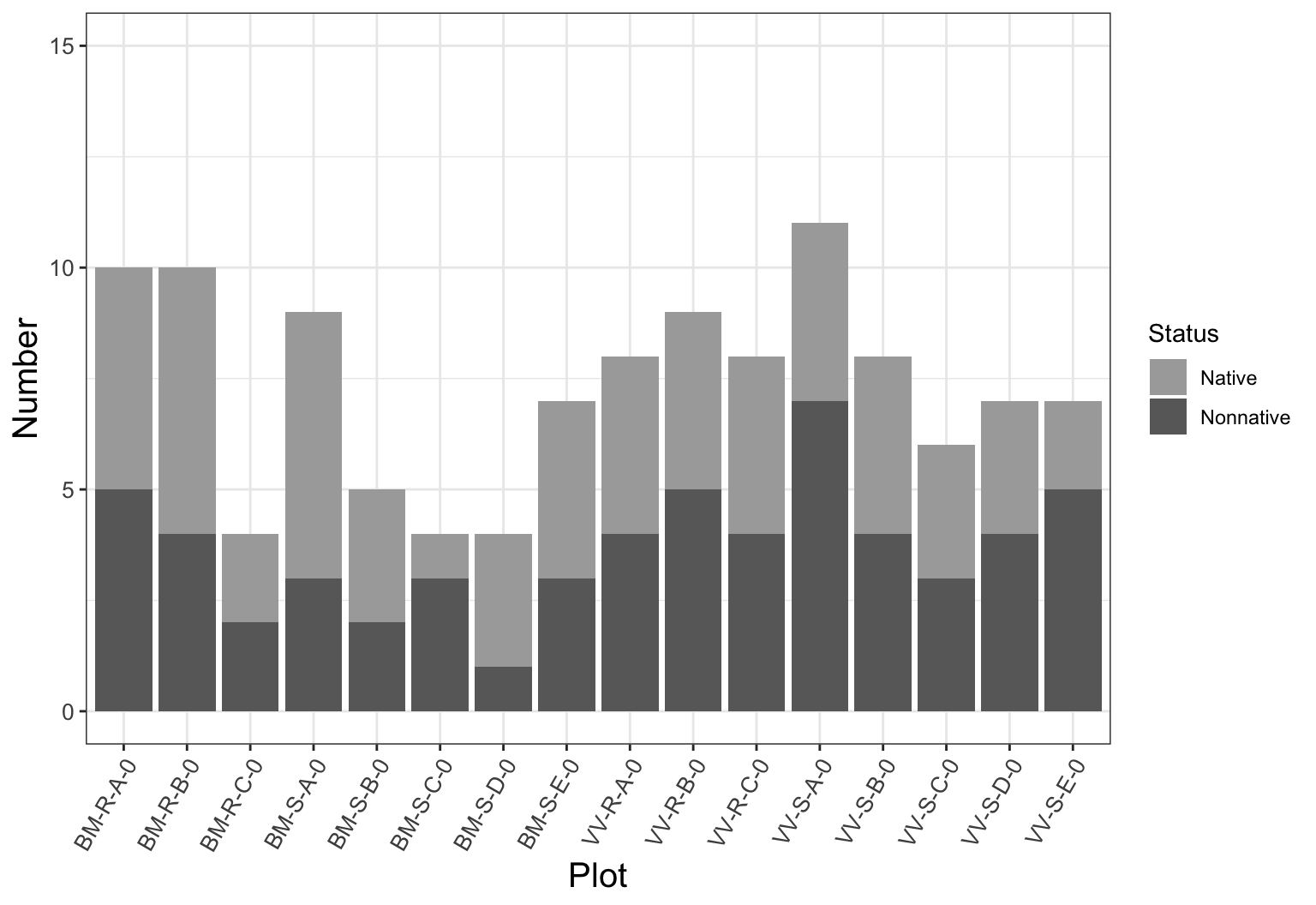


**I**


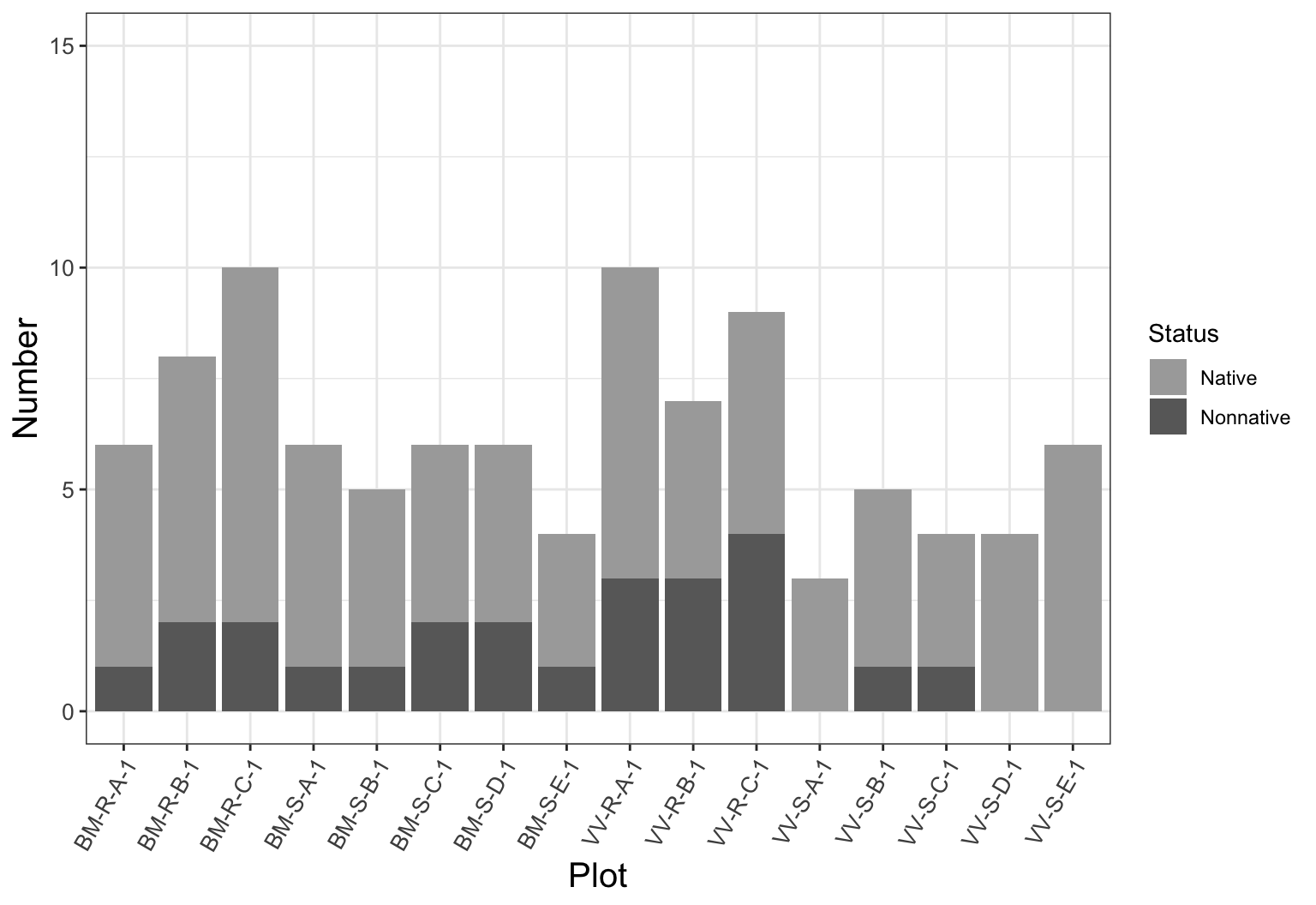


**IV**


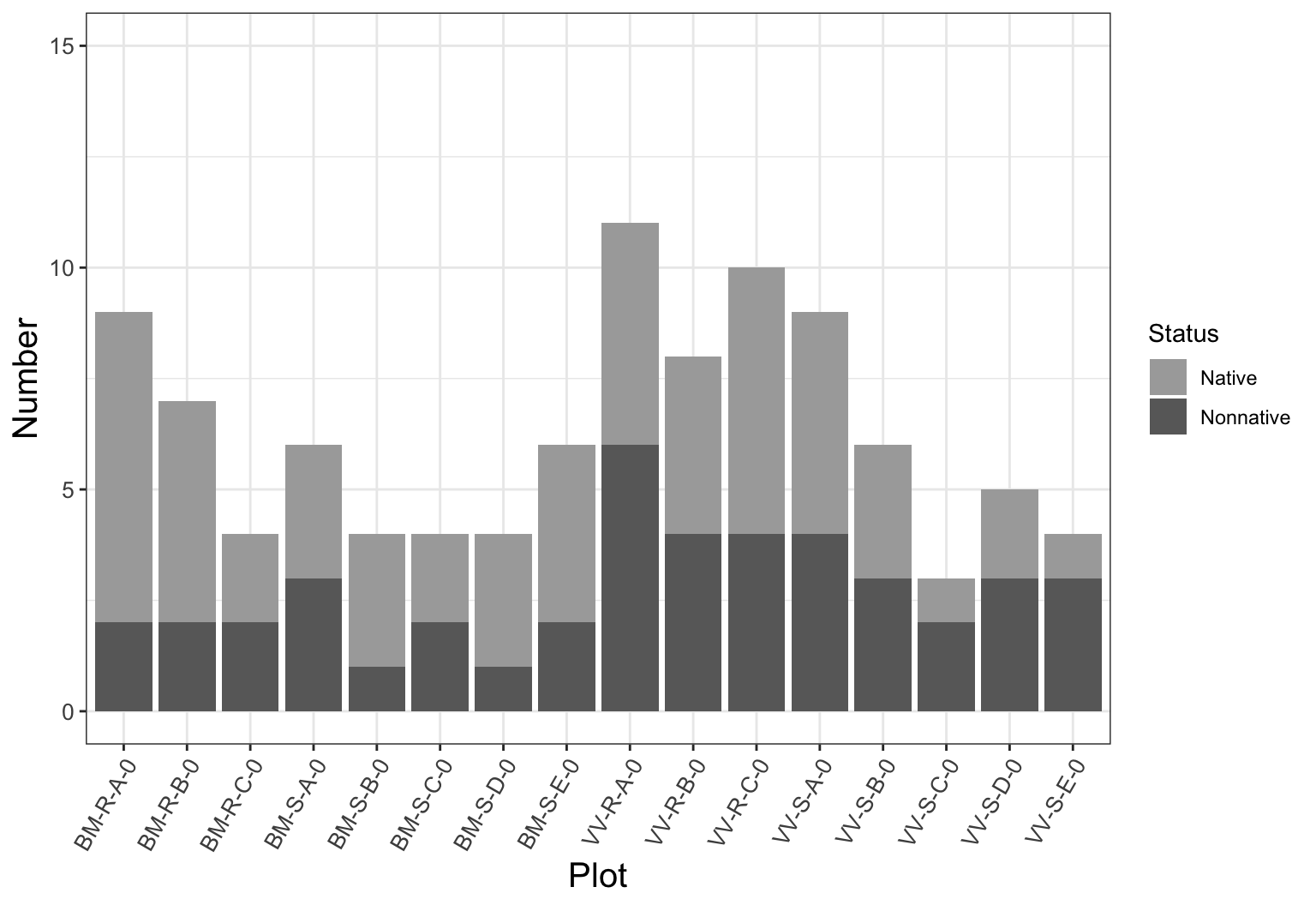


**II**


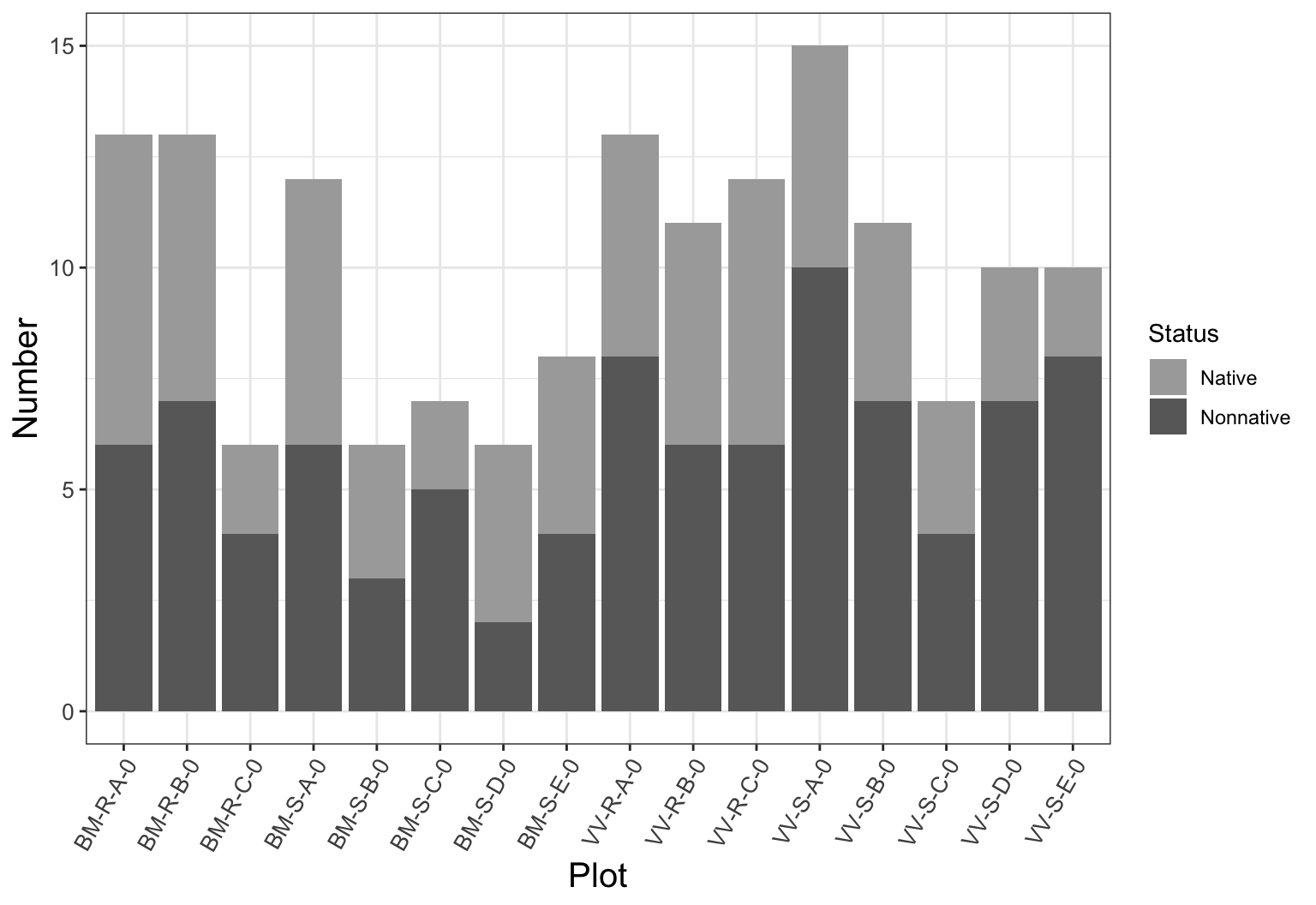


**Fig C. Native status of species in germination plots.**

(I) Removal plots. (II) Topsoil plots. (III) Topsoil plots, removing species germinated in greenhouse. (IV) Topsoil plots, removing species only appearing in topsoil. (II), (III) and (IV) suggest that the commercial topsoil used for capping is a source of nonnative species.


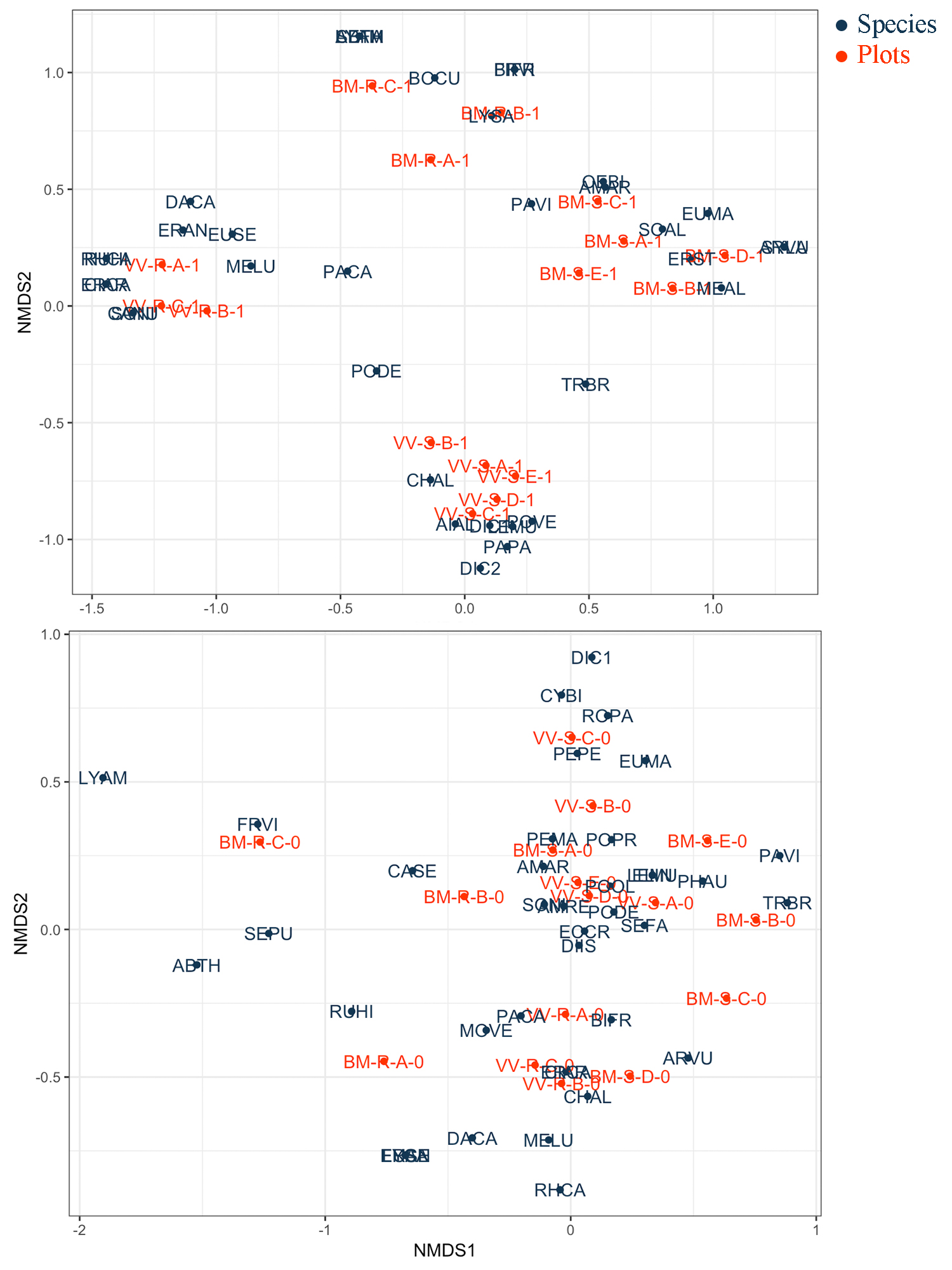


**I**

**II**

**Fig D.** **NMDS results of site difference, showing both plots and species.**

(I) Removal plots. (II) Topsoil plots. Plots are in red and species are in dark blue. See **Table** **H** for associated statistical analyses.

*Functional Trait Analysis Results*

Four clusters, one graminoid (cluster 4), two forbs (1 and 3), and one miscellaneous with shrubs, trees, some forbs and graminoids (2) were derived from the distance matrix (**Fig E**). Clustering analyses based on the two distance matrices both showed robust patterns of clustering with very similar species composition. The Hopkins statistic ranged from 0.20 to 0.30, implying valid clustering.

**Fig E. Functional trait clustering for all species in germination plots.**

**
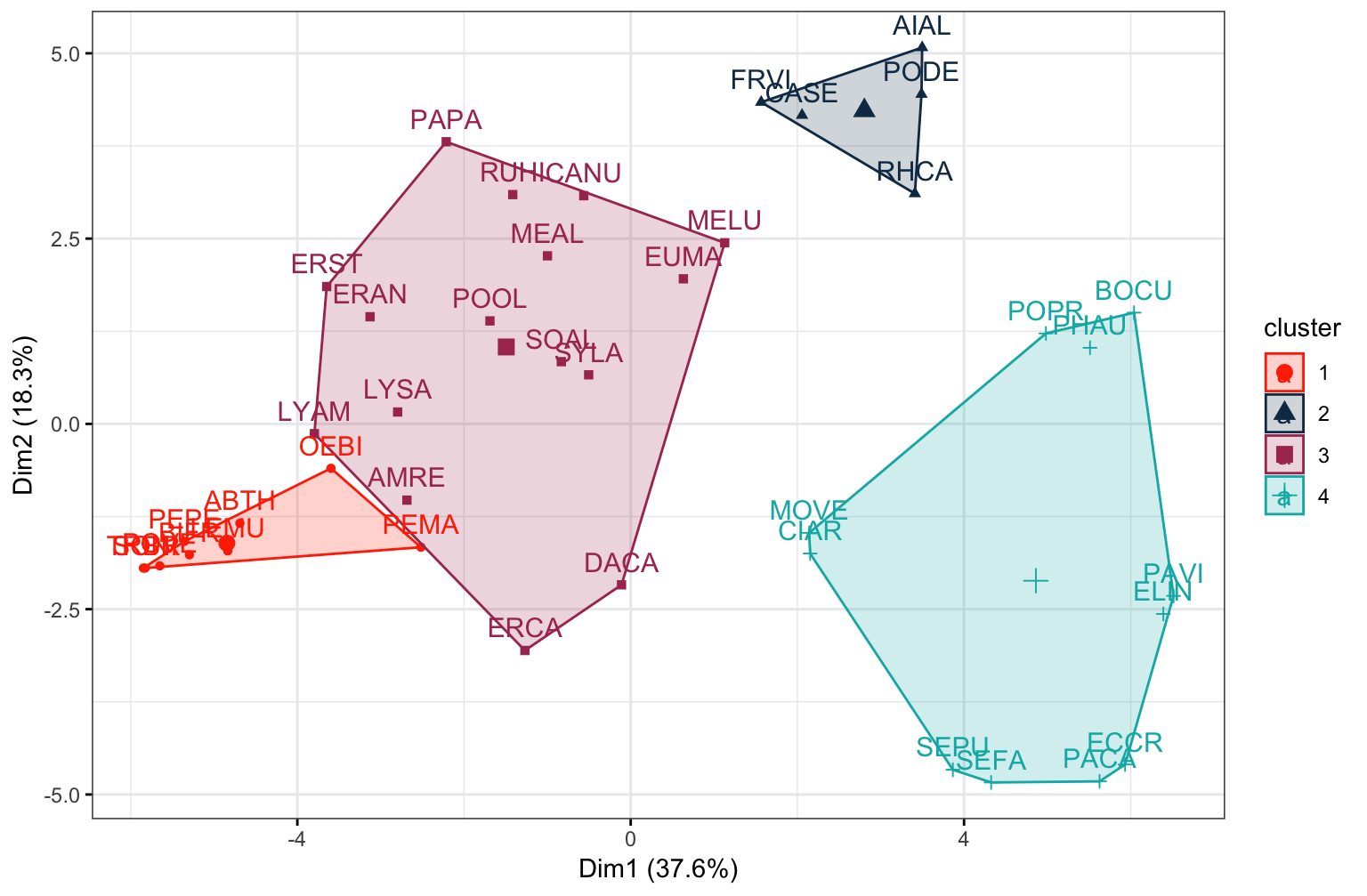
**

**Supporting Information References**

Dray S., and A. Dufour. 2007. The ade4 package: implementing the duality diagram for ecologists. *Journal of Statistical Software*, 22(4), 1-20.

Dray, S., and P. Legendre. 2008. Testing the species traits-environment relationships: the fourth‐corner problem revisited. *Ecology* 89, no. 12: 3400-3412.

Grime, J. Philip, J. G. Hodgson, and R. Hunt. 2014. *Comparative Plant Ecology: A Functional*

*Approach to Common British Species*. Springer.

Freyman, W. A., L. A. Masters, and S. Packard. 2016. The Universal Floristic Quality

Assessment (FQA) Calculator: an online tool for ecological assessment and monitoring. *Methods in Ecology and Evolution* 7, no. 3: 380-383.

Galili, T. 2015. Dendextend: A R package for visualizing, adjusting and comparing trees of

hierarchical clustering. *Bioinformatics* 31, no. 22: 3718-3720.

Gower, J. C. 1971. A general coefficient or similarity and some or its properties. *Biometrics*

27: 857-8

Hilty, J. 2017. Illinois Wildflowers. Accessed Mar 4, 2019.

<https://www.illinoiswildflowers.info>

Kassambara, A., and F. Mundt. 2017. factoextra: extract and visualize the results of

multivariate data analyses. R package version 1.0.5.

<https://CRAN.Rproject.org/package=factoextra>

Kattge, J., S. Diaz, S. Lavorel, I. C. Prentice, P. Leadley, G. Bönisch, E. Garnier et al. 2011.

TRY–a global database of plant traits. *Global change biology* 17, no. 9: 2905-2935.

Laliberté, E., and P. Legendre. 2010. A distance-based framework for measuring functional diversity from multiple traits. *Ecology* 91:299-305.

Lee, Y. Y., and S. Ventura. 2017. lindia: automated linear regression diagnostic. R package

version 0.9. <https://CRAN.R-project.org/package=lindia>

Maechler, M., P. Rousseeuw, A. Struyf, M. Hubert, and K. Hornik. 2018. cluster: cluster

analysis basics and extensions. R package version 2.0.7-1.

Oksanen, J., F. G. Blanchet, M. Friendly, R. Kindt, P. Legendre, D. McGlinn, P. R. Minchin,

R. B. O'Hara, G. L. Simpson, P. Solymos, M. H. H. Stevens, E. Szoecs and H. Wagner. 2018. vegan: community ecology package. R package version 2.5-3.

<https://CRAN.R-project.org/package=vegan>

Pavoine, S., J. Vallet, A. Dufour, S. Gachet, and H. Daniel. 2009. On the challenge of treating

various types of variables: application for improving the measurement of functional diversity. *Oikos* 118, no. 3: 391-402.

Pinheiro J., D. Bates, S. DebRoy, D. Sarkar, R Core Team. 2018. nlme: linear and nonlinear

mixed effects models. R package version 3.1-137. <https://CRAN.R-project.org/package=nlme>

Simpson, G. L. 2017. ggvegan: ‘ggplot2’ plots for the ‘vegan’ package. R package version 0.0-

9.

Wickham, H. 2017. tidyverse: easily install and load the 'tidyverse'. R package version 1.2.1.

<https://CRAN.R-project.org/package=tidyverse>
